# Complete Genome and Methylome Sequences of Two *Salmonella enterica* spp.

**DOI:** 10.1128/genomeA.01599-15

**Published:** 2016-01-21

**Authors:** Kuan Yao, Tim Muruvanda, Richard J. Roberts, Justin Payne, Marc W. Allard, Maria Hoffmann

**Affiliations:** a Center for Food Safety and Nutrition, Division of Microbiology, Office of Regulatory Science, U.S. Food and Drug Administration, College Park, Maryland, USA; b School of Systems Biology, George Mason University, Manassas, Virginia, USA; c New England Biolabs, Inc., Ipswich, Massachusetts, USA

## Abstract

*Salmonella enterica* is responsible for major foodborne outbreaks worldwide. It can cause gastroenteritis characterized by diarrhea, vomiting, and fever. *Salmonella* infections raise public health concerns along with consequential economic impacts. In this report, we announce the first complete genome sequences of *Salmonella enterica* subsp. *enterica* serovar Choleraeuis (*S.* Choleraeuis) ATCC 10708 and *Salmonella enterica* subsp. *enterica* serovar Pullorum (*S*. Pullorum) ATCC 9120, isolated from patients with diarrhea.

## GENOME ANNOUNCEMENT

*Salmonella enterica* subsp. *enterica* serovar Choleraeuis (*S.* Choleraeuis) is a nontyphoid serotype that is highly swine-adapted and can be transmitted to humans by contaminated pork products and slaughter environments (1). According to the U.S. Centers for Disease Control and Prevention, the number of *S*. Choleraesuis infections has been gradually decreasing in the United States (2). Still, *S*. Choleraesuis infections remain prevalent in Asia. For example, *S*. Choleraesuis accounted for 5% of the reported *Salmonella* infections in northern Taiwan (3), and since 2008 between 85 and 163 cases associated with *S*. Choleraesuis infection have been reported each year in Japan (1).

*S. enterica* subsp. *enterica* serovar Pullorum (*S*. Pullorum) infects mainly poultry and wild birds and can lead to pullorum disease (4, 5). The strain persists in the reproductive tract of chickens, causing eggs and chicks to obtain the pathogen from vertical transmission. *S*. Pullorum is next to S. Gallinarum in being responsible for economic losses in poultry production. Given the importance of these serovars, we announce here the availability of complete closed reference genomes for *S.* Choleraeuis ATCC 10708 and *S*. Pullorum ATCC 9120.

Both isolates, *S*. Choleraesuis and *S*. Pullorum, were cultured in Trypticase soy broth (Becton, Dickinson, Franklin Lakes, NJ, USA) overnight at 37°C. The genomic DNA was extracted from overnight cultures using the DNeasy blood and tissue kit (Qiagen, Inc., Valencia, CA, USA). The genomes were sequenced using the Pacific Biosciences (PacBio) RS II sequencing platform, as previously reported (6, 7). The library was prepared based on the 20-kb PacBio sample preparation protocol and sequenced using P6/C4 chemistry on four single-molecule real-time (SMRT) cells with a 240-min collection time. The continuous long read (CLR) data were *de novo* assembled using the PacBio hierarchical genome assembly process (HGAP version 3.0) with default parameters (8). The assembled sequences were annotated using the NCBI Prokaryotic Genomes Annotation Pipeline and subsequently deposited at DDBJ/EMBL/GenBank.

The *S*. Choleraesuis genome was fully closed with 182× coverage for the chromosome and 51× coverage for the plasmid sequence. The chromosome consists of 4,824,322 bp with a GC content of 52.24%, while the plasmid comprises 119,113 bp with a GC content of 48%. PHAST (9) analysis identified four “intact” prophages, including *Enterobacteria* phage Fels-2, *Enterobacteria* phage P22, prophage Gifsy-2, and one unknown phage.

The *S*. Pullorum genome was fully closed with 355× coverage for the chromosome and 178× coverage for the plasmid. The genome size was 4,694,842 bp with a GC content of 52.19% and consisted of 4,630 genes. The plasmid was found to comprise 86,650 bp. PHAST analysis identified two “intact” prophages, including *Enterobacteria* phage Fels-2 and prophage Gifsy-2.

Sequencing of these isolates also measured the kinetic variations of nucleotide incorporation rates to infer DNA methyltransferase activities (10). The SMRT data of the methylomes were analyzed and are summarized in Table 1. They are also deposited in REBASE (11). DNA methyltransferase recognition motifs, which were detected by SMRT sequencing, and the genes encoding the various motifs can be found for *S*. Choleraesuis at http://rebase.neb.com/cgi-bin/pacbioget?16669 and for *S*. Pullorum at http://rebase.neb.com/cgi-bin/pacbioget?10067.

**TABLE 1:** Summary of active methylases and their recognition sequences

Strain	CAGAG	ATGCAT	CCWGG	GATC
*S*. Choleraesuis	M.Sen10708I	M.Sen10708II	M.Sen10708Dcm	M.Sen10708DamP^a^ M.Sen10708ORF5545P^b^**
*S*. Pullorum			M.Sen9120Dcm	M.Sen9120DamP^a^ M.Sen9120ORF17650P^b^

^a^ Probably active.

^b^ Probably inactive, as it is encoded on a prophage (*Enterobacteria* phage Fels-2, NC_010463).

### Nucleotide sequence accession numbers.

The complete genome and plasmid sequences of *S*. Choleraesuis are available in GenBank under the accession numbers CP012344 and CP012345. The complete chromosome and plasmid sequences of *S*. Pullorum were deposited under the accession numbers CP012347 and CP012348.
